# Management of sepsis in neutropenic cancer patients: 2018 guidelines from the Infectious Diseases Working Party (AGIHO) and Intensive Care Working Party (iCHOP) of the German Society of Hematology and Medical Oncology (DGHO)

**DOI:** 10.1007/s00277-019-03622-0

**Published:** 2019-02-22

**Authors:** Matthias Kochanek, E. Schalk, M. von Bergwelt-Baildon, G. Beutel, D. Buchheidt, M. Hentrich, L. Henze, M. Kiehl, T. Liebregts, M. von Lilienfeld-Toal, A. Classen, S. Mellinghoff, O. Penack, C. Piepel, B. Böll

**Affiliations:** 1 Department I of Internal Medicine, Faculty of Medicine and University Hospital Cologne, University of Cologne, Kerpener Str. 62, 50937 Cologne, Germany; 2 Intensive Care in Hematologic and Oncologic Patients (iCHOP), Cologne, Germany; 3 Department of Hematology and Oncology, Medical Center, Otto-von-Guericke University Magdeburg, Magdeburg, Germany; 4 Medical Department III, University Medical Center & Comprehensive Cancer Center Munich, Munich, Germany; 5 Department for Hematology, Hemostasis, Oncology and Stem Cell Transplantation Hannover Medical School, Hannover, Germany; 6 Department of Hematology and Oncology, Mannheim University Hospital, Mannheim, Germany; 7 Department of Medicine III – Hematology and Oncology, Red Cross Hospital, Munich, Germany; 8 Department of Medicine, Clinic III – Hematology, Oncology, Palliative Medicine, Rostock University Medical Center, Rostock, Germany; 9 Department of Internal Medicine I, Clinic Frankfurt (Oder), Frankfurt, Germany; 10 Department of Bone Marrow Transplantation, West German Cancer Center, University Hospital Essen, University of Duisburg-Essen, Essen, Germany; 11 Department for Hematology and Medical Oncology, University Hospital Jena, Jena, Germany; 12 Department for Hematology, Oncology and Tumorimmunology, Campus Virchow Clinic, Charité Universitätsmedizin Berlin, Berlin, Germany; 13 Department of Hematology, Oncology and Infectious Diseases, Klinikum Bremen-Mitte, Bremen, Germany

**Keywords:** Guideline, Sepsis, Septic shock, Cancer, Neutropenia, Management

## Abstract

Sepsis and septic shock are major causes of mortality during chemotherapy-induced neutropenia for malignancies requiring urgent treatment. Thus, awareness of the presenting characteristics and prompt management is most important. Improved management of sepsis during neutropenia may reduce the mortality of cancer therapies. However, optimal management may differ between neutropenic and non-neutropenic patients. The aim of the current guideline is to give evidence-based recommendations for hematologists, oncologists, and intensive care physicians on how to manage adult patients with neutropenia and sepsis.

## Introduction

Sepsis and septic shock are leading causes for intensive care unit (ICU) admission and mortality in patients with hematologic malignancies or solid tumors undergoing intensive cytotoxic chemotherapy [1, 2]. Therefore, optimization of screening, diagnostic procedures, and management of sepsis may improve outcome of neutropenic hematologic and oncologic patients with sepsis. These guidelines are a revised version of the 2013 Guidelines of the Infectious Diseases Working Party (AGIHO) and Intensive Care in Hematologic and Oncologic Patients (iCHOP) Working Party of the German Society of Hematology and Medical Oncology (DGHO) that should be helpful in daily clinical use.

With many new cancer treatment options, neutropenia may not soon be seen as frequently. Thus, sepsis and septic shock in patients with neutropenia may not be so prominent. Instead, corresponding changes in the immune system of patients through specific cancer therapies show a completely different infection response and thus other septic processes. Therefore, infections and sepsis in cancer patients should always be monitored from this point of view.

The guidelines were thematically divided into 26 subtopics according to the new sepsis 2016 guidelines [3]. This allows for direct comparability with the general sepsis guidelines and identifies specific differences immediately. The guideline preparation was sometimes very difficult as only few studies for neutropenic septic patients were published. Because of this, many results have been transferred. Therefore, more studies should be conducted in the future for this particular patient group, or even better not exclude neutropenic septic patients from studies.

Subcommittees of 2–4 experts in the field of infectious diseases in hematology and oncology and intensive care specialists were responsible for literature search in one of the subtopics. We systematically searched Medline for English language publications up to March 2018 using the key terms: sepsis, neutropenia (and similar terms), and one of the following: bacteremia, bloodstream infection, screening, definition, epidemiology, incidence, risk factors, prognosis, treatment, antibiotic, antifungal, cardiovascular, pulmonary failure, ventilation, mechanical ventilation, renal dysfunction, renal failure, renal replacement therapy, dialysis, hemofiltration, nutrition, hyperglycemia, steroid, coagulation, growth factor, immunoglobulin, transfusion, venous thromboembolism prophylaxis, stress ulcer prophylaxis, and setting of goals of care. The consensus process was performed as an email- and meeting-based discussion group.

The level of evidence and the strength of recommendation of particular treatment options are weighed and graded according to predefined scales, as outlined in Table 1 [4].

**Table 1:** Strength of recommendation (SoR) and quality of evidence (QoE)

Strength of recommendation (SoR)		
Grade A	Strongly supports a recommendation for use	
Grade B	Moderately supports a recommendation for use	
Grade C	Marginally supports a recommendation for use	
Grade D	Supports a recommendation against use	
Quality of evidence (QoE)		
Level I	Evidence from at least one properly designed randomized, controlled trial	
Level II	Evidence from at least one well-designed clinical trial, without randomization; from cohort or case-controlled analytic studies (preferably from > 1 center); from multiple time series; or from dramatic results of uncontrolled experiments	Added index for Level II: • r: meta-analysis or systematic review of randomized controlled trials • t: transferred evidence, that is, results from different patients’ cohorts, or • Similar immune-status situation • h: comparator group is a historical control • u: uncontrolled trial • a: published abstract (presented at an international symposium or meeting)
Level III	Evidence from opinions of respected authorities, based on clinical experience, descriptive case studies, or reports of expert committees	

After preparing the tables for the recommendations (including “strength of recommendation, SoR” and “quality of evidence, QoE”), they were peer-reviewed by the AGIHO review committee on March 23, 2018. The final version of the manuscript was prepared by the corresponding author and has been approved by all authors.

There was no industry input into the guidelines. No member of the guidelines committee received honoraria for any role in the guidelines process.

## Pathophysiology

Currently, sepsis is one of the leading causes of death in neutropenic cancer patients in ICUs. The host response to systemic infection is heterogeneous and influenced by clinical and biological factors, making the pathogenic processes of sepsis and its outcome highly variable. Immunocompromised states are associated with lower sepsis survival, with neutropenia presenting a particularly high risk for critically ill patients with sepsis [5]. As the neutrophils are believed to have a central role in the pathogenesis of sepsis and related organ dysfunction, distinct clinical or molecular characteristics remain to be investigated in neutropenic patients with sepsis.

Despite many prospective and retrospective analyses, no significant pathophysiological difference between neutropenic and non-neutropenic patients could be identified. In a study by Reilly et al., it could be shown that neutropenia was independently associated with a higher risk for acute kidney injury (AKI) and was characterized by a profile of high interleukin-6, interleukin-8, and granulocyte colony-stimulating factors (G-CSF) relative to non-neutropenic sepsis [6]. Evidently, Toll-like receptor (TLR) expression and polymorphism also seem to play a role in the development of sepsis. In patients with neutropenic fever, levels of mRNA expression of TLR2 and TLR4 were significantly higher in sepsis patients compared to patients without sepsis [7]. Further, polymorphisms of TLR2 and TLR4 influence the risk of infectious complications in patients with acute myeloid leukemia undergoing chemotherapy [8]. However, it should be emphasized that changes in TLR expression and in polymorphism also result in an altered immune response in non-neutropenic patients [9]. Sepsis has been described as an inflammatory response. The improved understanding of immunosuppression indicates, however, that sepsis initiates a more complex immunologic response varying over time and entailing pro- and anti-inflammatory mechanisms such as the activation of various checkpoints [10, 11].

## Definition

A formal definition of sepsis has always been the subject of intense debate. We suggest using the diagnostic consensus criteria for sepsis: the Third International Consensus Definitions for Sepsis and Septic Shock (Sepsis-3, 2016) define sepsis as a life-threatening organ dysfunction caused by a dysregulated host immune response to infection [12].

**Fig. 1:**
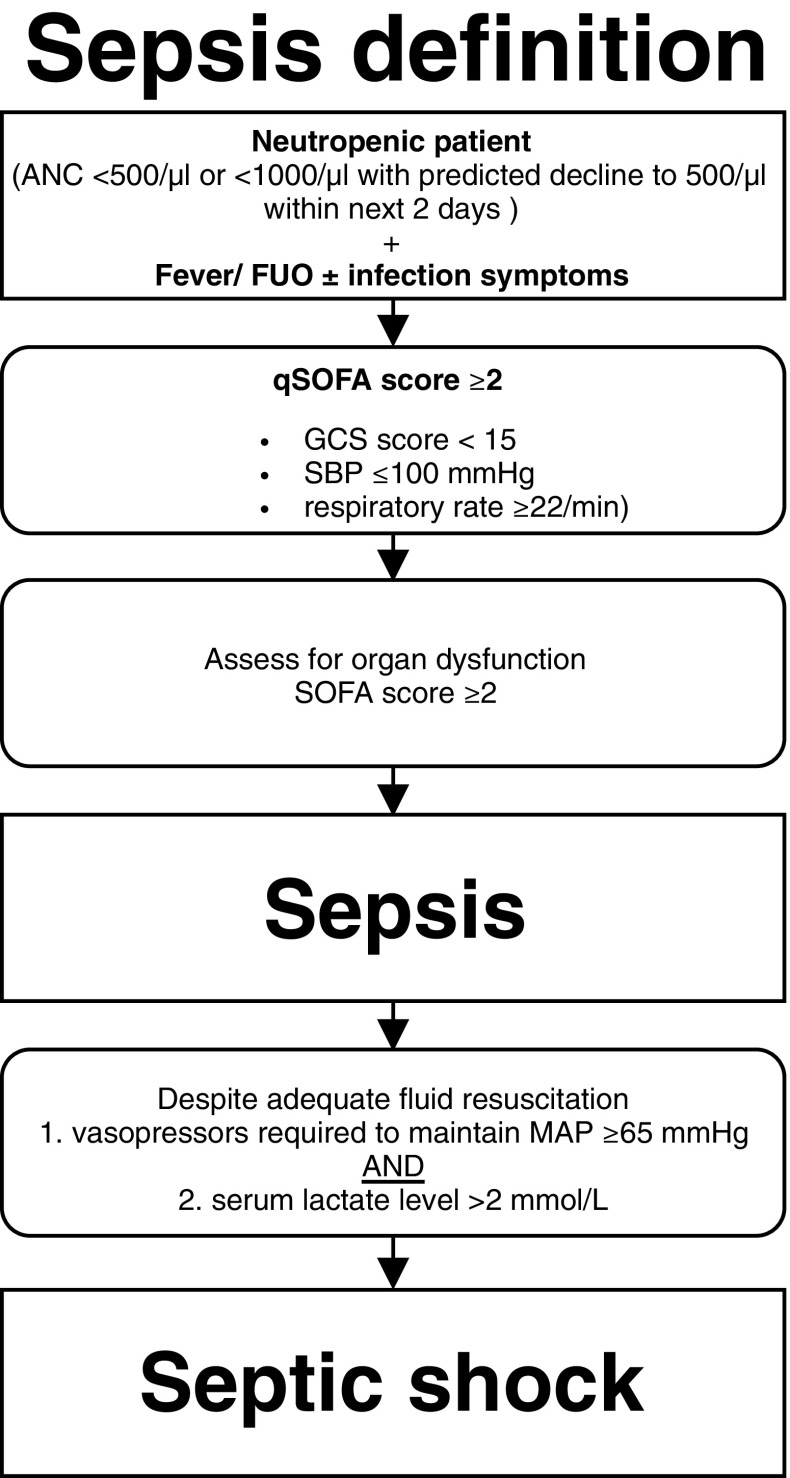
Sepsis definition for neutropenic patients analog to Sepsis-3 guidelines [12]. (ANC, absolute neutrophil count; FUO, fever of unknown origin; qSOFA, quick SOFA; SOFA, Sepsis-Related Organ Failure Assessment; GCS, Glasgow Coma Scale; SBP, systolic blood pressure; MAP, mean arterial pressure)

Due to the lack of evidence that definition criteria should be defined differently in neutropenic patients, we have agreed on the following diagram in analogy to the Sepsis-3 definition criteria (Fig. 1). Considering new therapeutic options, therapies that interfere with the function of neutrophils, and not just the neutrophil count alone, should be crucial.

However, there are few limitations. Both the quick Sepsis-Related Organ Failure Assessment (qSOFA) score and especially the Sepsis-Related Organ Failure Assessment (SOFA) score cannot be used without restrictions in neutropenic patients: (i) mental status may change independently from the onset of sepsis and assessment is therefore sometimes limited. (ii) Tumor-associated symptoms or complications can lead to neurological deficits. (iii) Platelet count cannot be used due to chemotherapy-associated or tumor-related thrombocytopenia, and (iv) chemotherapy-induced elevation of bilirubin and creatinine may influence the SOFA score calculation.

Two large recently published meta-analyses estimating mortality show a poor sensitivity for the qSOFA score (0.51 and 0.60) but a higher specificity (0.83 and 0.72) while the old Systemic Inflammatory Response Syndrome (SIRS) criteria show a high sensitivity (0.86 and 0.88) but a poor specificity (0.25 and 0.29) [13, 14]. The newly introduced qSOFA score certainly facilitates bedside screening for sepsis by the simple, compressed, and fast calculation. However, negative qSOFA score (< 2) should not cause any delays in starting treatment for sepsis if there is clinical suspicion in this high-risk population of neutropenic patients.

It should be emphasized that this is the *definition* of sepsis. The diagram is not suitable for *diagnosis* of sepsis.

## Incidence/proportion

There are no reliable data on the overall incidence of sepsis and septic shock in patients with neutropenia due to the different statistical methods used in individual studies. Furthermore, the majority of studies did not use uniform definitions for sepsis and often included non-neutropenic patients or focused on distinct patient subgroups.

Proportion of sepsis or septic shock in patients with neutropenia ranges from 7 to 45% depending on the study and publication year (Table 2).

**Table 2:** Proportion of neutropenic septic patients

Author, year of publication	Study title	Study period	Number of centers	Included patients (*n*)	Neutropenic patients *n* (%)
Soares 2006 [15]	Prognosis of critically ill patients with cancer and acute renal dysfunction	2000–2004	1	309	37 (12)
Soares 2008 [16]	Short- and long-term outcomes of critically ill patients with cancer and prolonged ICU length of stay	2000–2005	1	1090	81 (7)
Soares 2010 [17]	Characteristics and outcomes of patients with cancer requiring admission to intensive care units: a prospective multicenter study	2007	28	717	52 (7)
Oeyen 2013 [18]	Long-term outcomes and quality of life in critically ill patients with hematological or solid malignancies: a single center study	2008–2009	1	483	32 (7)
Azoulay 2013 [1]	Outcomes of critically ill patients with hematologic malignancies: prospective multicenter data from France and Belgium	2010–2011	17	1011	289 (29)
Lee 2015 [19]	Effect of early intervention on long-term outcomes of critically ill cancer patients admitted to ICUs	2010–2012	1	525	237 (45)

The studies included patients with underlying hematological or oncological disease, neutropenic patients, or patients with cancer in general. Remarkably, most of the evaluations were conducted in hospitals specialized in the treatment of neutropenic cancer patients with sepsis. Therefore, estimates on the epidemiology of sepsis and septic shock in patients with neutropenia beyond specialized academic centers are lacking.

## Risk factors associated with neutropenic sepsis

There is a variety of risk factors for the development of sepsis in neutropenic patients. The evaluations of the studies distinguish between risk factors for infections that trigger sepsis and direct risk factors for sepsis.

### Factors for bacteremia/infection

Numerous studies in cancer patients show that neutropenia itself is an independent risk factor for infection and/or bacteremia. There is a significant higher rate of bacteremia and infection, potentially resulting in sepsis, in patients with severe neutropenia (absolute neutrophil count < 500/μL) and in those with neutropenia duration longer than 7 days. Additionally, acute leukemia, a prolonged hospital stay, prior surgery, advanced disease, delay of ICU admission, a Hickman catheter, or pre-treatment with antibiotics or chemotherapy were significantly associated with bloodstream infection and sepsis and septic shock in patients with neutropenia in hematological and oncological patients [1, 20–23].

### Factors for development of sepsis and septic shock

In a previous study, factors associated with the occurrence of severe sepsis (definition according to the guidelines of 2012 [24]) were as follows: hypophosphatemia (< 0.8 mmol/L), hypoproteinemia (< 62 g/L), and non-adapted antibiotic therapy at the onset of severe sepsis (*p* = 0.019) [25]. The development of septic shock in febrile neutropenia is independently predicted by the presence of pneumonia, tachypnea, increased serum levels of procalcitonin (PCT) (≥ 1.5 ng/mL), high lactate levels (> 3 mmol/L), decreased serum levels of bicarbonate (< 17 mmol/L), antithrombin (< 70%), or factor VIIa (< 0.8 ng/mL). A low Multinational Association for Supportive Care in Cancer (MASCC) risk index score of < 21 is associated with an increased risk for septic shock in febrile neutropenic patients [1, 2, 25–31].

## Initial resuscitation

There is no evidence that sepsis and septic shock in patients with neutropenia need to be treated differently to non-neutropenic patients according to the sepsis guidelines 2016 (AIII) [3].

To date, no studies have provided any evidence for differences in the treatment of sepsis and septic shock in patients with neutropenia compared to non-neutropenic septic patients. In a retrospective single center study, Schnell et al. found an equal duration and dose of norepinephrine given to neutropenic patients compared to non-neutropenic patients [32]. In a meta-analysis of 3 large Early Goal Directed Therapy (EGDT) trials of septic patients, nearly 15% of 3737 included patients were immunosuppressed. Although there was no information on the proportion of patients with neutropenia, outcomes between the EGDT group and the usual care group were comparable [33]. In summary, all studies listed have a small but significant proportion of either cancer patients, immunosuppressed patients, or neutropenic patients. Therefore, although neutropenia is an independent risk factor for increased mortality, there is no evidence to support any differences in the initial resuscitation of neutropenic septic patients [34–36]. In a recent update of the Surviving Sepsis Campaign Bundles, the urgency of the initial diagnosis and treatment steps was once again emphasized [37]. The former 3- or 6-h bundles were all determined for the first hour. Five goals to be targeted within the first hour were explicitly mentioned: measurement of the lactate level, blood cultures, administration of a broad-spectrum antibiotic, rapid fluid administration, and possibly vasopressors to maintain blood pressure due to septic shock patients. No specific patient subgroups were excluded from these recommendations to be treated differently.

Not only in this chapter but throughout the guidelines, it is striking that there are no reasons to treat patients with sepsis in neutropenia differently than non-neutropenic septic patients. Nevertheless, neutropenic patients are excluded in all large randomized trials. In the future, there are no reasons to exclude neutropenic patients from sepsis studies.

## Screening criteria for sepsis and performance improvement

Before ICU admission, the treatment goals and the prognosis should be identified. No patients should be admitted to the ICU against their wishes (AIII).

Patients in neutropenia and signs or symptoms of infection should be screened for sepsis daily (AIII).

A structured checklist diagnosis is not possible, but the treating physician must decide clinically whether the patient is septic or not (AIII).

Early warning scoring system (EWS) and early involvement of intensive care teams on hemato-oncology ward is recommended (AIIht) [38].

In clinically unclear situations, a neutropenic patient should be admitted early to an ICU. Neutropenia should be used as triage criterion in cancer patients considered for ICU admission (AIIr).

In critically ill neutropenic cancer patients, ICU admission should not be delayed if indicated. (AIIt) [39–41].

If a patient has been admitted to the ICU, daily meetings between oncologists and intensivists for care planning and implementation of protocols are recommended (AIIt) [2].

In cancer patients who develop sepsis or septic shock during neutropenia, the patient’s will and prognosis should be determined before transfer to the ICU. Admission to an ICU against the will of the patient is not indicated and unethical.

Sepsis screening has been associated with decreased mortality [42]. The implementation of a core set of recommendations (bundle) has been a cornerstone of programs improving sepsis performance and management. There is no doubt that this also applies to neutropenic patients. In a retrospective study, Bokhari et al. showed that an EWS system and early involvement of the ICU team can improve mortality of hematology patients (20% were neutropenic patients with sepsis) [38]. The following clinical parameters have been included in the EWS system: respiratory rate, heart rate, temperature, systolic blood pressure, central nervous system, and estimated hourly urine output.

The prognostic impact of neutropenia for sepsis and septic shock remains controversial. In an unselected population of neutropenic critically ill patients, several recent studies failed to demonstrate any impact of neutropenia, neutropenia duration, or resolution of neutropenia on the outcome of critically ill cancer patients [1, 43, 44]. In a large meta-analysis, it was demonstrated in more than 1700 neutropenic critically ill patients that neutropenia was independently associated with poor outcome despite a meaningful survival [45]. Nevertheless, certain infections together with neutropenia are associated with extremely poor outcome or high mortality (e.g., *Fusarium* infection [46]). However, neutropenic septic patients do not provide a typical clinical picture. Therefore, the likelihood of sepsis should be checked during daily clinical visits. In a retrospective study, Soares et al. demonstrated that daily visits with oncologists and intensivists for care planning and implementation of protocols (sepsis campaign bundles, sepsis guidelines, etc.) were associated with lower hospital mortality in critically ill cancer patients [2].

If the interdisciplinary team decides to transfer the patient to an ICU, this should be done immediately. Several studies that included a significant number of patients with neutropenia have shown that the timely admission of cancer patients to the ICU improves survival [31, 40, 41].

The use of the qSOFA score as a screening method for the identification of sepsis, as propagated in the Sepsis-3 definition, is being discussed vigorously not only for non-neutropenic patients among experts [12] (see also “Definition,” definition of sepsis).

## Diagnosis

There is no evidence that septic neutropenic patients differ to non-neutropenic septic patients according to the sepsis guidelines 2016 (AIII) [3].

Neutropenic cancer patients with a suspicion or proof of an infection should be screened for signs of acute organ dysfunction(s) daily (AIII).

Biomarkers can be used to support the diagnosis of bacterial/fungal infections but are unable to confirm or rule out an infection (BIIu-BIII).

Modified multiplex PCR protocols might be used to support the diagnosis of infection leading to sepsis (CIIu).

**Fig. 2:**
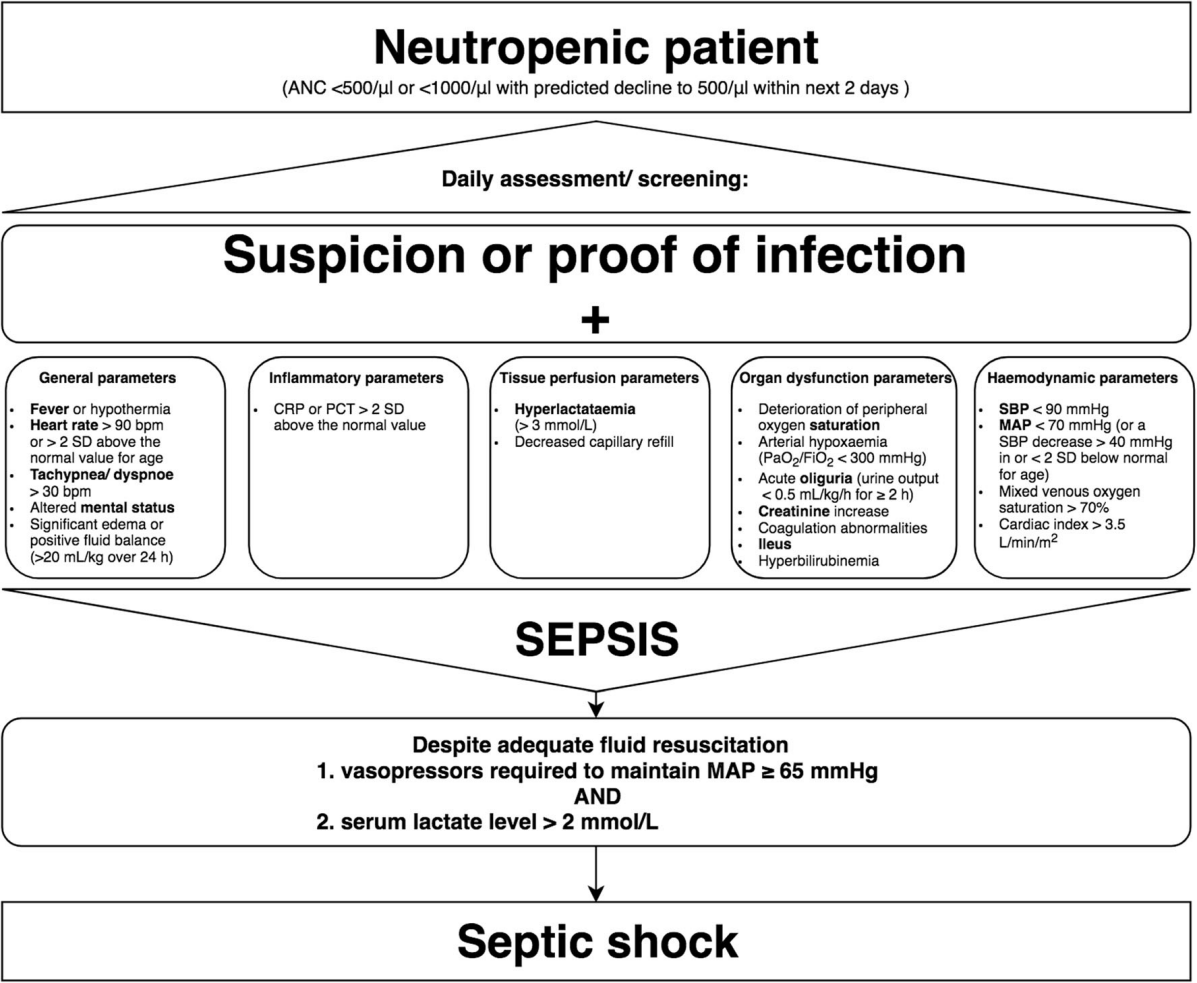
Diagram for diagnosis of sepsis and septic shock. Important clinical symptoms are highlighted in bold. ANC, absolute neutrophil count; SBP, systolic blood pressure; MAP, mean arterial pressure; bpm, beats per minute; SD, standard deviation; CRP, C-reactive protein; PCT, procalcitonin

A diagnostic algorithm is outlined in Fig. 2.

It must be emphasized that the diagnosis of sepsis in neutropenic patients is difficult to make and largely depends on the experience of the treating physician. As already stated in the interdisciplinary consensus statement of the DGHO, Austrian Society of Hematology and Oncology (OeGHO), German Society for Medical Intensive Care Medicine and Emergency Medicine (DGIIN), and Austrian Society of Medical and General Intensive Care and Emergency Medicine (ÖGIAIN), timely recognition, diagnostic steps, and rapid therapy initiation are of decisive importance for the prognosis of critical ill cancer patients [47]. Thus, early identification of patients at risk for critical deterioration seems crucial. Severity of illness scores (e.g., qSOFA score, SOFA score) can be used for describing groups of patients or estimate mortality. Therefore, those scores should not be used for individual diagnosis or for the decision for ICU admission, but can help to identify neutropenic septic patients [12–14].

Every neutropenic patient should have a daily screening of an experienced physician. There was no intentional evaluation of the individual parameters for the clinical decision-making. However, some points were highlighted to speed up the diagnosis. There are a variety of studies that have tested different inflammatory markers in cancer or neutropenic patients such as PCT, CRP, and IL-6. These inflammatory markers may be helpful for the diagnosis of sepsis, but normal values do not rule out sepsis [48–54]. Modified multiplex PCR protocols to recognize pathogens in blood samples causing sepsis might improve the diagnosis of sepsis [55].

## Antimicrobial therapy

Empirical antimicrobial treatment using anti-pseudomonal broad-spectrum antibiotics must be started immediately in neutropenic patients with sepsis (AIIrt).

We recommend initial treatment with piperacillin/tazobactam or meropenem or imipenem/cilastatin (AIII).

A combination treatment with an aminoglycoside may be considered in neutropenic patients with septic shock (BIII).

In case of clinically stabilizing patients or detection of pathogens sensitive to ß-lactam, it is recommended to stop the aminoglycosides (AIII).

Risk factors for invasive fungal infections and/or for uncontrolled cardiopulmonary instability, an antifungal therapy should be considered (AIII).

A large retrospective study including more than 2000 patients showed that effective antimicrobial administration within the first hour of documented hypotension is associated with increased survival from severe sepsis [56]. In this study, each hour of delay in antimicrobial administration over the ensuing 6 h was associated with an average decrease in survival of 7.6% [56].

In neutropenic patients with sepsis, results from randomized controlled trials are lacking, and recommendations are based on study results from non-neutropenic patients as well as on expert opinions [57].

Fever of unknown origin in patients with neutropenia should be treated as recommended in the AGIHO guidelines [58]. In case of sepsis or septic shock, we recommend initial treatment with piperacillin/tazobactam or with meropenem or imipenem/cilastatin. Knowledge of local epidemiology and resistance patterns is crucial for the choice of antimicrobial agents. Importantly, colonization with resistant bacteria must be considered.

Meta-analyses show that a combination treatment with aminoglycosides increased renal toxicity without improving efficacy in neutropenic patients with bacteremia [58–60]. However, in a retrospective study, the use of β-lactam antibiotic/aminoglycoside combinations was associated with superior outcome, as compared with single-agent antimicrobial treatment, in neutropenic patients with septic shock [56]. Another retrospective study showed reduced hospital mortality in non-neutropenic patients with severe bacterial sepsis after combination therapy comprising at least two antibiotics of different mechanisms versus antibiotic monotherapy [61]. Taken together, a combination treatment with an aminoglycoside may be considered in neutropenic patients with septic shock (BIII).

Clinicians should also consider whether fungal species are likely pathogens when choosing initial therapy. Risk factors for invasive fungal infections include diabetes mellitus, chronic liver failure, chronic renal failure, prolonged invasive vascular devices (hemodialysis catheters, central venous catheters), total parenteral nutrition, recent major surgery (particularly abdominal), prolonged administration of broad-spectrum antibiotics, prolonged hospital/ICU admission, recent fungal infection, severe skin and soft tissue infections, and multisite fungal colonization. Even if uncontrolled cardiopulmonary instability continues to develop, antifungal therapy should be considered. However, there are no well-controlled studies [62].

Of note, the current guidelines refer to further AGIHO guidelines and recommendations for antimicrobial therapy of neutropenic cancer patients and hematopoietic stem cell transplant (HSCT) recipients, as listed in Table 3. The guidelines are regularly re-evaluated and published.

**Table 3:** Overview of current AGIHO guidelines and recommendations for antimicrobial therapy of neutropenic cancer patients and HSCT recipients

Source of infection	AGIHO guidelines reference
Central venous catheter–related infections	[63]
CNS infections	[64]
Fever of unknown origin (FUO)	[65]
Gastrointestinal complications	[66]
Infections in allogeneic HSCT	[67]
Infections in autologous HSCT	[68]
Invasive fungal infections	[62]
Lung infiltrates	[69]

*HSCT*, hematopoietic stem cell transplantation; *CNS*, central nervous system

## Source control

There is no evidence that source control is different in septic neutropenic patients than non-neutropenic patients according to the sepsis guidelines 2016 (AIII) [3].

A source control (e.g., surgery or CT-controlled puncture) should be done as soon as possible (AIIt).

If possible, all intravascular devices should be removed in case of suspected infection (AIIt).

There is no evidence that source control in neutropenic patients should be any different to that in non-neutropenic patients [3]. Neutropenic patients often have central venous access, which should be removed in case of suspected infection at the onset of sepsis.

Other infections should be addressed according to their location or accessibility. It is important to ensure that, if possible, the least invasive procedure is used.

However, surgical interventions should not be delayed because of accompanying pancytopenia. Instead, blood products should be transfused as needed.

## Fluid therapy

There is no evidence that septic neutropenic patients need to be treated differently than non-neutropenic patients according to the sepsis guidelines 2016 (AIII) [3].

Due to a lack of studies in neutropenic septic patients, recommendations of the 2016 sepsis guideline should be adopted.

## Vasoactive medications

There is no evidence that septic shock in patients with neutropenia needs to be treated differently than non-neutropenic patients according to the sepsis guidelines 2016 (AIII) [1].

There is no indication that neutropenic septic patients should be treated otherwise. It should be noted that many patients develop heart failure due to multiple prior cancer treatments. The incidence of heart failure is dose-dependent, for example, in doxorubicin > 48% [70]. Therefore, screening for concomitant cardiac insufficiency should be performed as part of catecholamine therapy.

## Corticosteroids

There is no evidence that septic shock in patients with neutropenia needs to be treated differently than non-neutropenic patients according to the sepsis guidelines 2016 (AIII) [3].

The continuation of a cortisone therapy should be evaluated individually (AIII).

The discussion about the use of corticosteroids in septic shock has been around for a long time and results from two high-ranking studies from 2018 were inconclusive [71, 72].

According to a recent meta-analysis by Rochwerg et al. that included all major studies of recent years [73], the use of corticosteroids may result in a small reduction in mortality in critically ill patients with septic shock but increases the risk of neuromuscular weakness. However, information about the number of included immunosuppressed or neutropenic patients was not provided in any of the studies.

Therefore, the use of corticosteroids can only be recommended analogously to non-neutropenic patients according to the sepsis guidelines 2016 [3].

Hematologic and oncological patients with preexisting cortisone therapy and sepsis and septic shock in patients with neutropenia are frequently transferred to the ICU where it must be evaluated individually whether cortisone therapy should be continued or not. Long-term cortisone therapy above the Cushing threshold may need to be slowly reduced during course of treatment.

There are some studies showing that invasive candidiasis could be caused by an immune reconstitution syndrome (dysregulation of Th1/ Th17 and Th2/ T_reg_ response) [74, 75]. Post-chemotherapy neutropenia in patients with hematological malignancy leads to an anti-inflammatory environment, particularly due to a Th2/ T_reg_ dominant response. It has been suggested that hepatosplenic candidiasis could in fact be an invasive fungal disease-related immune reconstitution syndrome. In a small retrospective study with 10 patients, it could be shown that the use of corticosteroids has a positive effect. In an update by the Infectious Diseases Society of America (IDSA) of the guideline for the management of candidiasis (2016) is given the following recommendation: for patients who have debilitating persistent fevers, short-term (1–2 weeks) treatment with nonsteroidal anti-inflammatory drugs, or corticosteroids can be considered (weak recommendation; low-quality evidence) [76].

## Blood products

There is no evidence that sepsis and septic shock in patients with neutropenia require different treatment than non-neutropenic patients according to the sepsis guidelines 2016 (AIII) [3].

Red blood cell (RBC) transfusion: we recommend that RBC transfusion occurs only when hemoglobin concentration decreases to < 7.0 g/dL (4.3 mmol/L) in adults in the absence of particular circumstances, such as myocardial ischemia, severe hypoxemia, or acute hemorrhage. No RBC transfusions should be performed for hemoglobin concentrations ≥ 7 g/dL (4.3 mmol/L) in the absence of risk factors (DIIt).

Granulocytes transfusion: there is low-grade evidence that patients do not benefit from therapeutic granulocyte transfusions in terms of clinical resolution of infection (CIII).

Platelet transfusion: in the absence of other risk factors for bleeding prophylactic, platelet transfusions should be given using the standard trigger level (≤ 10 × 10^9^/L) (BI-IItr).

For neutropenic septic patients or prior to an intervention with an increased risk of bleeding, platelet transfusions should be indicated individually (AIII).

In a retrospective analysis of a prospectively collected database, Mirouse et al. showed in septic patients with hematological malignancies (with or without neutropenia) that RBC transfusion within the first 2 days was associated with higher mortality at day 7 (20.5 vs. 13.3%, *p* = 0.02), higher in-ICU mortality (39 vs. 25.2%, *p* < 0.001), and in-hospital mortality (51 vs. 36.6%, *p* < 0.001). RBC transfusion remained independently associated with increased in-hospital mortality in multivariate logistic regression (OR 1.52, *p* = 0.03) and propensity score-adjusted (OR 1.64, *p* = 0.03) analysis. Although only one third of the patients examined were neutropenic, one can assume that a conservative transfusion strategy should be used [77].

Similarly, a subgroup analysis of patients with severe comorbidity treated in the Transfusion Requirements in Septic Shock (TRISS) trial showed no benefit of liberal versus restrictive transfusion strategies in patients with hematological malignancy (*p* = 0.47). However, all included hematological patients were analyzed without focus on neutropenia [78].

Transfusions of granulocytes have a long history of usage in neutropenic patients with severe infections. The Cochrane analysis by Estcourt et al. gives a good overview of the studies carried out until 2016. In addition to many case reports and small studies, 10 randomized trials were included in the evaluation. However, only neutropenic patients with severe infections were evaluated but not those with sepsis [79]. There is insufficient evidence to determine whether granulocyte transfusions affect all-cause mortality and low-grade evidence indicating that therapeutic granulocyte transfusions do not increase the number of patients with clinical resolution of an infection. The latest large randomized study from 2015 showed that there was no overall effect of granulocyte transfusion on the primary outcome (composite of survival plus microbial response at 42 days after randomization) [80]. Again, no septic neutropenic patients were studied, only patients with neutropenia and severe infections. Within the review committee, 10 voted in favor, 10 voted undetermined, and 5 voted against the recommendation.

The current practice of administering prophylactic platelet transfusions using the standard trigger level (≤ 10 × 10^9^/L) in the absence of other risk factors for bleeding is reasonable [81]. To date, no study has shown any benefit of a higher trigger threshold for septic patients. However, platelet transfusion should be decided on an individual patient basis, in particular in patients who are scheduled for intervention with a potential risk of bleeding.

## Hematopoietic growth factors

We do not recommend the routine additional use of G-CSF or GM-CSF to standard treatment of sepsis and septic shock in patients with neutropenia (DI-IIr).

G-CSF–induced neutropenia recovery carries a risk of respiratory status deterioration with acute lung injury or acute respiratory distress syndrome (ARDS) (DIII).

G-CSF and GM-CSF increase the number of circulating granulocytes in the blood. This property was the rationale to use both growth factors to treat infections in addition to antibiotics in febrile patients with chemotherapy-induced neutropenia. A Cochrane analysis of 15 randomized controlled trials including a total of 1553 patients showed that G-CSF/GM-CSF effectively reduces the time to neutrophil recovery and the length of hospitalization [82]. Of note, the use of a CSF plus antibiotics in individuals with chemotherapy-induced febrile neutropenia had no effect on overall mortality. However, this Cochrane analysis evaluated the influence of G-/GM-CSF on chemotherapy-induced neutropenia and did not specifically focus on neutropenic septic patients. Although the routine use of these two growth factors in patients without infection is generally well tolerated, there are an increasing number of publications on respiratory deterioration with ARDS during G-CSF/GM-CSF–induced neutropenia recovery [83–87].

## Immunoglobulins

There is marginally degree of evidence to support the use of intravenous immunoglobulins (IVIG) in sepsis and septic shock in patients with neutropenia (CIIrt).

Pathophysiological considerations suggest that the administration of IVIG may be beneficial in patients with severe infection or sepsis. However, a randomized controlled trial in neutropenic cancer patients with sepsis or septic shock as well as a meta-analysis, a large retrospective study and a Cochrane analysis—the latter mainly including non-neutropenic septic patients—did not show significant differences in survival between patients treated with or without IVIG [88–91].

In contrast, three additional meta-analyses found an overall reduction of mortality with the use of IVIG in non-neutropenic septic patients.

However, given the significant heterogeneity among the trials, all authors emphasize the need of larger well-designed and adequately powered clinical trials [90–92]. In patients with relevant (and known) antibody deficiency treatment with IVIG is recommended.

## Blood purification

Cytokine adsorption cannot be recommended at this time for sepsis and septic shock in patients with neutropenia (DIIt-III).

To date, there are an increasing number of publications dealing with the topic of blood purification as a treatment option for severe septic patients. However, due to small number of patients included in the studies, different blood purification systems, and different study protocols, no firm conclusions can be drawn. Notably, neutropenic septic patients were excluded from all studies so far. The expert group does therefore not recommend the use of blood purification in neutropenic septic patients outside clinical studies.

## Anticoagulants

There are no studies supporting the use of heparin in sepsis and septic shock in patients with neutropenia (DIII).

There is insufficient evidence to support antithrombin substitution in any category of critically ill participants including the subset of patients with sepsis and disseminated intravascular coagulation (DIItr).

Neither heparin nor antithrombin or thrombomodulin has been studied in neutropenic septic patients. Also, in the sepsis guideline 2016, there is no recommendation for heparin and thrombomodulin along with a recommendation against use of antithrombin. Thus, the use of anticoagulants cannot be recommended for neutropenic septic patients.

## Mechanical ventilation

There is no evidence that sepsis and septic shock in patients with neutropenia need to be treated differently than non-neutropenic patients according to the sepsis guidelines 2016 (AIII) [3].

Noninvasive ventilation (NIV) should not be used in patients with a respiratory failure and a PaO_2_/FiO_2_ lower than 150 mmHg (DIIt).

NIV did not improve survival compared to oxygen only (AIIrt).

High-flow nasal cannula (HFNC) oxygen when compared with standard oxygen did not reduce intubation or survival rates and may be used in special circumstances (AIIt).

Prone position is recommended for patients with severe ARDS (BIItr).

Acute respiratory failure (ARF) is the most common organ dysfunction in hematologic patients. Despite significant improvements over time, it is still associated with high mortality rates of greater than 50% in patients requiring invasive mechanical ventilation (IMV) for severe ARF [1].

NIV is used in acute exacerbated chronic pulmonary disease or in acute cardiac pulmonary edema. However, the role of NIV is not well documented in hypoxic ARF. Studies from 2001 that included selected immunosuppressed patients with pneumonitis and ARF showed that early initiation of NIV was associated with significant reductions in the rates of endotracheal intubation and serious complications with improved likelihood of survival to hospital discharge [92]. The 2015 published guidelines of the German Society of Pneumology and Ventilatory Medicine recommend a NIV trial in immunosuppressed (hemato-oncological) patients to avoid intubation [93]. In view of this recommendation, however, it is important to point out contraindications and criteria for NIV termination as well as intubation criteria. Several recent studies revealed limitations of NIV for immunocompromised patients. A meta-analysis of 2380 mainly hematologic non-septic patients showed that NIV as initial ventilation strategy was associated with lower mortality. Importantly, 61% (range 40 to 78%) of patients experienced NIV failure with secondary intubation, which itself was associated with increased mortality [94]. In a large multicenter observational investigation in 1004 cancer patients with ARDS, NIV failure occurred in 71% of patients and was, again, independently associated with mortality, while mortality of ARDS patients undergoing IMV per se had decreased to 52% in recent years [95]. A recent large multicenter randomized study compared the use of NIV to conventional oxygen in immunocompromised (mainly hematologic; no information about neutropenia) patients with hypoxic ARF (acute cardiogenic pulmonary edema or hypercapnia, i.e., PaCO_2_ > 50 mmHg excluded, pre-specified intubation criteria) [96]. Early NIV compared with oxygen therapy alone did not reduce 28-day mortality. However, study power was limited.

HFNC oxygen therapy, which has been widely used in recent years, has shown advantages in the rate of intubation and mortality compared to conventional oxygen therapy or NIV in non-immunosuppressed and immunosuppressed patients [97, 98].

In a multinational observational prospective cohort study across 16 countries (68 centers), Azoulay et al. found [99] that in immunocompromised patients (17% neutropenic patients/without sepsis) with hypoxemic ARF HFNC oxygen therapy has a positive effect on intubation but not on mortality rates. Use of NIV did not impact outcomes either. The need for intubation was associated with mortality with higher odds for mortality in case of NIV or HFNC oxygen therapy failure.

The same group has published a study comparing HFNC oxygen therapy with conventional oxygen therapy in a post hoc analysis of a randomized controlled trial of NIV in critically ill immunocompromised patients (neutropenia included) with hypoxemic ARF [100]. Disappointingly, HFNC oxygen therapy, when compared with standard oxygen, did not reduce intubation or survival rates.

In case of using one of these procedures, an escalation from conventional oxygen therapy to HFNC oxygen therapy and NIV or vice versa should be avoided. Failure of any of these strategies should immediately (no improvement within 3–6 h; risk factors Table 4) lead to IMV. An unnecessary delay in intubation is associated with increased mortality and should be avoided.

**Table 4:** Risk factors for NIV failure in cancer patients

Prior to NIV	Vasopressor need Multiple organ failure Airway involvement by malignancy Acute respiratory distress syndrome Unknown etiology of ARF Delayed onset of ARF
During NIV	Patient not tolerating NIV No improvement of ABG within 3–6 h Respiratory rate > 30/min NIV dependency ≥ 3 days Clinical or respiratory deterioration Unknown etiology of ARF

*NIV*, noninvasive ventilation; *ARF*, acute respiratory failure; *ABG*, arterial blood gas analysis

## Sedation, analgesia, and physiotherapeutic prophylaxis

There is no evidence that sepsis and septic shock in patients with neutropenia need to be treated differently than non-neutropenic patients according to the sepsis guidelines 2016 (AIII) [3].

A strategy for whole-body physiotherapy—consisting of interruption of sedation and physical and occupational therapy in the earliest days of critical illness—is recommended (AIII).

The use of standardized weaning protocols is recommended (AIItr).

Sedation and analgesia as part of IMV should be kept as short and as small as possible. The expert group agrees that especially in neutropenic septic patients weaning should be carried out as soon and effectively as possible. Pancytopenia induced by chemotherapy and muscle atrophy, which is often induced by cortisone therapy, should be treated by physiotherapeutic and ergotherapeutic measures.

## Glucose control

There is no evidence that sepsis and septic shock in patients with neutropenia need to be treated differently than non-neutropenic patients according to the sepsis guidelines 2016 (AIII) [3].

In concomitant cortisone therapy for treatment of hemato-oncological diseases blood glucose levels should be closely monitored.

## Management of renal dysfunction—renal replacement therapy

There is no evidence that sepsis and septic shock in patients with neutropenia need to be treated differently to non-neutropenic patients according to the sepsis guidelines 2016 (AIII) [3].

Given the lack of good quality studies, no other recommendation can be made.

Potentially nephrotoxic drugs should be avoided if not necessarily indicated, and all drugs require renal dosage adjustments. A tumor lysis syndrome should be early recognized and treated.

## Bicarbonate therapy

There is no evidence that septic shock in patients with neutropenia needs to be treated differently than non-neutropenic patients according to the sepsis guidelines 2016 (AIII) R [3].

A small study that included 20 patients with acute leukemia and sepsis or septic shock with neutropenia [29] showed that decreased levels of bicarbonate were associated with a significant increase in mortality. However, a lowered level of bicarbonate may merely express the severity of the septic shock. Thus, it remains unclear whether substituting bicarbonate has any benefit.

Treatment with bicarbonates for tumor lysis syndrome should be avoided, which may increase the risk of calcium-phosphate crystals deposition [101].

## Venous thromboembolism prophylaxis

We recommend pharmacologic prophylaxis with unfractionated heparin or low-molecular-weight heparin for venous thromboembolism prophylaxis in the absence of contraindications (AIItr).

The recommendations are transferred from non-neutropenic patients. There are no studies investigating thromboembolism prophylaxis in neutropenic ICU patients even in the presence of thrombocytopenia [102].

## Stress ulcer prophylaxis

There is no evidence that sepsis and septic shock in patients with neutropenia need to be treated differently than non-neutropenic patients (AIII) [3].

## Nutrition

There is no evidence that sepsis and septic shock in patients with neutropenia need to be treated differently than non-neutropenic patients (AIII) [3].

In patients with severe neutropenic enterocolitis, severe viral or bacterial gastrointestinal infections, or severe gastrointestinal graft-versus-host disease (GVHD), enteral nutrition should be paused or, at least, carried out with caution (AIII).

Concomitant cancer-associated cachexia should not result in hyperalimentation (AIII).

Enteral nutrition is preferred over parenteral nutrition, because it is associated with a lower rate of infections (BIIt).

Cachexia is a multifactorial and multi-organ syndrome that is one of the main causes of morbidity and mortality in late stages of chronic conditions such as acquired immunodeficiency syndrome, chronic obstructive pulmonary disease, congestive heart failure, multiple sclerosis, tuberculosis, and cancer. In patients with cancer the incidence is highest in gastric and pancreatic cancer (∼ 80%) while the frequency is markedly lower in breast cancer and leukemia (∼ 40%) [103]. Concomitant cancer-associated cachexia should not result in hyperalimentation.

There are no studies on nutrition in neutropenic septic patients. Basically, there are the same conditions and prerequisites for cachexia as in non-neutropenic septic patients. Most recommendations reported are extrapolated from analyses in critically ill and well-nourished patients without neutropenia. Enteral nutrition is preferred over parenteral nutrition unless contraindicated or impossible, as it is associated with a lower rate of infection. The calculation of the calorie requirement depends on the sepsis severity and time course and does not differ from non-neutropenic septic patients.

As there is insufficient evidence for the use of probiotics in septic neutropenic patients [104], its use is not recommended. In patients undergoing allogeneic HSCT, the neutropenic diet did not offer a protective effect against infection [105]. However, a study on the role of neutropenic diet has not been performed in septic neutropenic patients.

The panel of experts agrees that in patients with severe neutropenic enterocolitis, severe viral or bacterial gastrointestinal infections, or severe gastrointestinal GVHD, enteral nutrition should be paused or, at least, carried out with great caution.

## Setting goals of care

Treatment goals and the short- and long-term prognosis of intensive care should be discussed with the patient and the relatives before admission to the ICU (AIII).

Full-code ICU management (without limitations of ICU resources) should be offered to all critically ill cancer patients if long-term survival may be compatible with the general prognosis of the underlying malignancy (AIIu).

Immediately after admission to ICU, we recommend discussing care and prognosis goals with patients and families (AIII).

There should be a daily exchange between intensive care physician and oncologists (AIII).

Therapy goals and prognosis must be re-evaluated daily and communicated to the relatives (AIII).

Possibilities for a better comprehension and therapy goal finding should be offered to the intensive care team and/or relatives. This includes printed information, structured communication, palliative care consultation, ethics consultation, or the use of structured family conferences conducted by the usual ICU team (BIII).

At any time point, all issues discussed must be communicated throughout the whole intensive care team. Differences in therapy goals should be recognized and discussed in the team (AIII).

Treatment in an ICU is a massive burden for patients as well as for relatives. Physicians should therefore discuss indications, possibilities but also dangers, and disadvantages of intensive care treatment with the patient and the relatives.

For a long time, cancer patients were not admitted to ICU due to their poor prognosis. Patients with neutropenia and sepsis were considered very critically. But especially neutropenic septic patients benefit from a timely and targeted therapy. Typically, full-code management (without limitations of ICU resources) applies to patients with curative therapeutic options and those in remission of their malignancy, as well as to patients in whom cure is not likely but the expected life span is substantial [1, 106, 107]. It has been suggested in an earlier consensus that an assumed prognosis of 1 year may be used as cutoff for clinical decision-making with regard to full-code status [108].

The authors of the above mentioned interdisciplinary DGHO/OeGHO/DGIIN/ÖGIAIN consensus statement believe this number is arbitrary and may be regarded as basis for individual considerations only [47].

After admission to the ICU, the treatment goals and options should be discussed with the patient and relatives. All advantages and disadvantages of the therapy should be communicated openly. Therapy limits must also be displayed transparently (Table 5).

**Table 5:** Summary of recommendations

Topic	Recommendations	SoR/QoE
Section Initial resuscitation	There is no evidence that sepsis and septic shock in patients with neutropenia need to be treated differently to non-neutropenic patients according to the sepsis guidelines 2016.	AIII
Section Screening criteria for sepsis and performance improvement	Before admission to the ICU, the treatment goals and the prognosis should be identified. No patients should be admitted to the intensive care unit against their wishes.	AIII
	Patients in neutropenia and signs or symptoms of infection should be screened for sepsis daily.	AIII
	A structured checklist diagnosis is not possible, but the treating physician must decide clinically whether the patient has sepsis.	AIII
	Early warning scoring system and early involvement of intensive care teams on hemato-oncology ward is recommended.	AIIht
	In clinically unclear situations, a neutropenic patient should be admitted early to an ICU. Neutropenia should be used as triage criterion in cancer patients considered for ICU admission.	AIIr
	In critically ill neutropenic cancer patients, ICU admission should not be delayed if indicated.	AIIt
	If a patient has been admitted to the ICU, daily meetings between oncologists and intensivists for care planning and implementation of protocols are recommended.	AIIt
Section Diagnosis	There is no evidence that sepsis and septic shock in patients with neutropenia differ to non-neutropenic septic patients according to the sepsis guidelines 2016.	AIII
	Neutropenic cancer patients with a suspicion or proof of an infection should be screened for signs of acute organ dysfunction(s) daily.	AIII
	Biomarkers can be used to support the diagnosis of bacterial/fungal infections but are unable to confirm or rule out an infection.	BIIu-BIII
	Modified multiplex PCR protocols might be used to support the diagnosis of an infection leading to sepsis.	CIIu
Section Antimicrobial therapy	Empirical antimicrobial treatment using anti-pseudomonal broad-spectrum antibiotics must be started immediately in neutropenic patients with sepsis.	AIIrt
	We recommend initial treatment with piperacillin/tazobactam or meropenem or imipenem/cilastatin.	AIII
	A combination treatment with an aminoglycoside may be considered in neutropenic patients with septic shock.	BIII
	In case of clinically stabilizing patients or detection of pathogens sensitive to ß-lactam, it is recommended to stop the aminoglycosides.	AIII
	Risk factors for invasive fungal infections and/or for uncontrolled cardiopulmonary instability, an antifungal therapy should be considered.	AIII
Section Source control	There is no evidence that source control is different in septic neutropenic patients than non-neutropenic patients according to the sepsis guidelines 2016.	AIII
	A source control (e.g., surgery or CT-controlled puncture) should be done as soon as possible.	AIIt
	If possible, all intravascular devices should be removed in case of suspected infection.	AIIt
Section Fluid therapy	There is no evidence that septic neutropenic patients need to be treated differently to non-neutropenic patients according to the sepsis guidelines 2016.	AIII
	Balanced crystalloids should be used for intravenous fluid administration.	AIIt
Section Vasoactive medications	There is no evidence that septic neutropenic patients need to be treated differently to non-neutropenic patients according to the sepsis guidelines 2016.	AIII
Section Corticosteroids	There is no evidence that septic shock in patients with neutropenia needs to be treated differently than non-neutropenic patients according to the sepsis guidelines.	AIII
	The continuation of a cortisone therapy should be evaluated individually.	AIII
Section Blood products	There is no evidence that sepsis and septic shock in patients with neutropenia require different treatment than non-neutropenic patients according to the sepsis guidelines 2016.	AIII
	Red blood cell (RBC) transfusion: we recommend that RBC transfusion occurs only when hemoglobin concentration decreases to < 7.0 g/dL (4.3 mmol/L) in adults in the absence of particular circumstances, such as myocardial ischemia, severe hypoxemia, or acute hemorrhage. No RBC transfusions should be performed for hemoglobin concentrations ≥ 7 g/dL (4.3 mmol/L) in the absence of risk factors.	DIIt
	Granulocytes transfusion: there is low-grade evidence that patients do not benefit from therapeutic granulocyte transfusions in terms of clinical resolution of infection.	CIII
	Platelet transfusion: in the absence of other risk factors for bleeding prophylactic, platelet transfusions should be given using the standard trigger level (≤ 10 × 10^9^/L).	BI-IItr
	For neutropenic septic patients or prior to an intervention with an increased risk of bleeding, platelet transfusions should be indicated individually.	AIII
Section Hematopoietic growth factors	We do not recommend the routine additional use of G-CSF or GM-CSF to standard treatment of sepsis and septic shock in patients with neutropenia.	DI-IIr
	G-CSF–induced neutropenia recovery carries a risk of respiratory status deterioration with acute lung injury or ARDS.	DIII
Section Immunoglobulins	There is marginally degree of evidence to support the use of intravenous immunoglobulins (IVIG) in sepsis and septic shock in patients with neutropenia.	CIIrt
Section Blood purification	Cytokine adsorption cannot be recommended at this time for sepsis and septic shock in patients with neutropenia.	DIIt-III
Section Anticoagulants	There are no studies supporting the use of heparin in sepsis and septic shock in patients with neutropenia.	DIII
	There is insufficient evidence to support antithrombin substitution in any category of critically ill participants including the subset of patients with sepsis and disseminated intravascular coagulation.	DIItr
Section Mechanical ventilation	There is no evidence that septic neutropenic patients need to be treated differently to non-neutropenic patients according to the sepsis guidelines 2016.	AIII
	NIV should not be used in patients with a respiratory failure and a PaO_2_/FiO_2_ lower than 150 mmHg.	DIIt
	NIV did not improve survival compared to oxygen only.	AIIrt
	High-flow nasal cannula (HFNC) oxygen when compared with standard oxygen did not reduce intubation or survival rates and may be used in special circumstances.	AIIt
	Prone position is recommended for patients with severe ARDS.	BIItr
Section Sedation and analgesia	There is no evidence that sepsis and septic shock in patients with neutropenia need to be treated differently than non-neutropenic patients according to the sepsis guidelines 2016.	AIII
	A strategy for whole-body physiotherapy—consisting of interruption of sedation and physical and occupational therapy in the earliest days of critical illness—is recommended.	AIII
	The use of standardized weaning protocols is recommended.	AIItr
Section Glucose control	There is no evidence that sepsis and septic shock in patients with neutropenia need to be treated differently than non-neutropenic patients according to the sepsis guidelines 2016.	AIII
Section Management of renal dysfunction—renal replacement therapy (RRT)	There is no evidence that sepsis and septic shock in patients with neutropenia need to be treated differently to non-neutropenic patients according to the sepsis guidelines 2016.	AIII
Section Bicarbonate therapy	There is no evidence that septic shock in patients with neutropenia needs to be treated differently than non-neutropenic patients according to the sepsis guidelines 2016.	AIII
Section Venous thromboembolism prophylaxis	We recommend pharmacologic prophylaxis with unfractionated heparin or low-molecular-weight heparin for venous thromboembolism prophylaxis in the absence of contraindications.	AIItr
Section Stress ulcer prophylaxis	There is no evidence that sepsis and septic shock in patients with neutropenia need to be treated differently than non-neutropenic patients.	AIII
Section Nutrition	There is no evidence that sepsis and septic shock in patients with neutropenia need to be treated differently than non-neutropenic patients.	AIII
	In patients with severe neutropenic enterocolitis, severe viral or bacterial gastrointestinal infections, or severe gastrointestinal graft-versus-host disease (GVHD), enteral nutrition should be paused or, at least, carried out with caution.	AIII
	Concomitant cancer-associated cachexia should not result in hyperalimentation.	AIII
	Enteral nutrition is preferred over parenteral nutrition, because it is associated with a lower rate of infections.	BIIt
Section Setting goals of care	Treatment goals and the short- and long-term prognosis of intensive care should be discussed with the patient and the relatives before admission to the intensive care unit.	AIII
	Full-code ICU management (without limitations of ICU resources) should be offered to all critical ill cancer patients if long-term survival may be compatible with the general prognosis of the underlying malignancy.	AIIu
	Immediately after admission to intensive care, we recommend discussing care and prognosis goals with patients and families.	AIII
	There should be a daily exchange between intensive care physician and oncologists.	AIII
	Therapy goals and prognosis must be re-evaluated daily and communicated to the relatives.	AIII
	Possibilities for a better comprehension and therapy goal finding should be offered to the intensive care team and/or relatives. This includes printed information, structured communication, palliative care consultation, ethics consultation, or the use of structured family conferences conducted by the usual ICU team.	BIII
	At any time point, all issues discussed must be communicated throughout the whole intensive care team. Differences in therapy goals should be recognized and discussed in the team.	AIII
